# Supplementary material for: End Sequence Analysis Toolkit (ESAT) expands the extractable information from single-cell RNA-seq data
Source: Genome Res. 2016 Oct;26(10):1397–410. doi: 10.1101/gr.207902.116 (PMC5052061; doi:10.1101/gr.207902.116)
Supplement: Supplemental Material [file supp_gr.207902.116_Supp_Materials.tar.gz › ESAT-esat_v0.1/doc/html/classbroad_1_1core_1_1motif_1_1misc_1_1_compute_conserved_motifs.html]

ESAT: broad.core.motif.misc.ComputeConservedMotifs Class Reference


ESAT


- Main Page
- Related Pages
- Packages
- Classes
- Files

- Class List
- Class Index
- Class Hierarchy
- Class Members

All Classes Namespaces Files Functions Variables Enumerator Pages

- broad
- core
- motif
- misc
- ComputeConservedMotifs

Public Member Functions |
Static Public Member Functions |
List of all members

broad.core.motif.misc.ComputeConservedMotifs Class Reference

|  |  |
| --- | --- |
| Public Member Functions | |
|  | ComputeConservedMotifs (Alignments region, String pwmFile, String saveDir, String alignDir, String alignFormat, File modelFile) throws Exception |
|  | |

|  |  |
| --- | --- |
| Static Public Member Functions | |
| static String[] | getMotifNames (String pwmFile) throws Exception |
|  | |
| static List< BED >[] | scoreMotifs (Alignments region, TreeScaler scaler, String alnFormat, String alignFile, String pwmFile, boolean reverseCompliment) throws Exception |
|  | |
| static void | main (String[] args) throws Exception |
|  | |

## Constructor & Destructor Documentation

|  |  |  |  |
| --- | --- | --- | --- |
| broad.core.motif.misc.ComputeConservedMotifs.ComputeConservedMotifs | ( | Alignments | *region*, |
|  |  | String | *pwmFile*, |
|  |  | String | *saveDir*, |
|  |  | String | *alignDir*, |
|  |  | String | *alignFormat*, |
|  |  | File | *modelFile* |
|  | ) |  | throws Exception |

Here is the call graph for this function:

Here is the caller graph for this function:

## Member Function Documentation

|  |  |  |  |  |  |
| --- | --- | --- | --- | --- | --- |
| static String [] broad.core.motif.misc.ComputeConservedMotifs.getMotifNames | ( | String | *pwmFile* | ) | throws Exception |

static

Here is the caller graph for this function:

|  |  |  |  |  |  |
| --- | --- | --- | --- | --- | --- |
| static void broad.core.motif.misc.ComputeConservedMotifs.main | ( | String[] | *args* | ) | throws Exception |

static

Here is the call graph for this function:

|  |  |  |  |
| --- | --- | --- | --- |
| static List<BED> [] broad.core.motif.misc.ComputeConservedMotifs.scoreMotifs | ( | Alignments | *region*, |
|  |  | TreeScaler | *scaler*, |
|  |  | String | *alnFormat*, |
|  |  | String | *alignFile*, |
|  |  | String | *pwmFile*, |
|  |  | boolean | *reverseCompliment* |
|  | ) |  | throws Exception |

static

Here is the caller graph for this function:

---

The documentation for this class was generated from the following file:

- java/broad/core/motif/misc/ComputeConservedMotifs.java


---

Generated on Fri Aug 15 2014 15:10:54 for ESAT by  

 1.8.7
